# The microbiology of bacterial peritonitis due to appendicitis in children

**DOI:** 10.1007/s11845-013-1055-2

**Published:** 2013-12-18

**Authors:** O. Obinwa, M. Casidy, J. Flynn

**Affiliations:** 1 Department of Surgery, Portiuncula Hospital, Ballinasloe, Ireland; 2 Department of Pathology, Portiuncula Hospital, Ballinasloe, Ireland

**Keywords:** Paediatric, Children, Appendicitis, Peritonitis, Antibiotic susceptibility

## Abstract

**Aim:**

The aim of this study was to investigate the microbiology of secondary bacterial peritonitis due to appendicitis and the appropriateness of current antimicrobial practice in one institution.

**Methods:**

A 14-year retrospective single-centre study of 69 consecutive paediatric patients (age 1–14 years) with appendicitis-related peritonitis and positive peritoneal specimen cultures was conducted. Post-operative outcomes, microbiology and antibiotic susceptibility of peritoneal isolates were analysed in all patients.

**Results:**

*Escherichia coli* was identified in 56/69 (81 %) peritoneal specimens; four isolates were resistant to amoxicillin–clavulanate, and one other isolate was resistant to gentamicin. Anaerobes were identified in 37/69 (54 %) peritoneal specimens; two anaerobic isolates were resistant to amoxicillin–clavulanate and one isolate was resistant to metronidazole. *Pseudomonas aeruginosa* was identified in 4/69 (6 %) peritoneal specimens, and all were susceptible to gentamicin. Streptococcal species (two Group F streptococci and three β-haemolytic streptococci) were identified in 5/69 (7 %) specimens, and all were susceptible to amoxicillin–clavulanate. Combination therapy involving amoxicillin–clavulanate and aminoglycoside is appropriate empirical treatment in 68/69 (99 %) patients. Addition of metronidazole to this regime would provide 100 % initial empirical coverage. Inadequate initial empiric antibiotic treatment and the presence of amoxicillin–clavulanate resistant *E. coli* were independent predictors of the post-operative infectious complications observed in 14/69 (20 %) patients.

**Conclusion:**

*E. coli* and mixed anaerobes are the predominant organisms identified in secondary peritonitis from appendicitis in children. Inadequate initial empirical antibiotic and amoxicillin–clavulanate resistant *E. coli* may contribute to increased post-operative infectious complications. This study provides evidence-based information on choice of combination therapy for paediatric appendicitis-related bacterial peritonitis.

## Introduction

Peritonitis is an inflammation of the membrane lining the inside of the abdomen/pelvis and all of the internal organs. Secondary peritonitis, as opposed to primary peritonitis, which occurs spontaneously, is the result of some other disease process [1]. In children, the most common cause of secondary peritonitis is perforated appendicitis and intra-abdominal abscess arising from acute appendicitis [1]. Secondary peritonitis in children is usually community acquired and accounts for prolonged hospitalisation [1, 2]. The aetiology of this disease is predominantly microbial with organisms from gut flora namely *Enterobacteriaceae* (coliforms) and anaerobes as pathogens [1, 3, 4].

Effective antimicrobials currently in use in Europe and throughout the world are fast losing ground as these causative pathogens, particularly the *Enterobacteriaceae*, acquire resistance to newly introduced antibiotics [5]. Despite this awareness, studies of secondary peritonitis due to appendicitis in children are limited [1, 6–9]. Treatment protocols vary widely and are guided by anti-microbial resistance patterns [7, 10, 11]. Complete antimicrobial coverage may be achieved using multiple agents [7, 10, 12–16]. However, targeted antibiotic treatment is preferable in the interest of decreasing resistance [1, 17–19].

Currently, in Ireland, antibiotic monotherapy (usually amoxicillin–clavulanate) is recommended for use in the management of uncomplicated appendicitis. For peritonitis, the recommendation is the use of combination antibiotic therapy involving amoxicillin–clavulanate and an aminoglycoside, gentamicin. In penicillin-sensitive patients, a combination of gentamicin or cephalosporin and metronidazole may be effective. The aim of this study was to investigate the microbiology of secondary bacterial peritonitis due to appendicitis and the appropriateness of current antimicrobial practice in one institution.

## Methods

A retrospective review of consecutive children (age between 1 and 14 years) presenting with secondary bacterial peritonitis due to appendicitis between January 1995 and December 2008 was conducted in one institution.

### Patients

There were 105 children with macroscopic findings of perforated appendicitis or abscess during appendicectomy during the study period. 25 (24 %) of these were excluded because no fluid specimens were sent for microbiological analysis and 11 (10 %) were further excluded because their peritoneal fluid specimens did not grow any organism. The remaining 69 children with perforated appendicitis and intra-abdominal abscess and who had positive cultures formed the principal cohort for analysis. It is important to note that subjects with simple acute non-perforated appendicitis or gangrenous appendicitis (macroscopic) without evidence of perforation were not part of this study.

### Specimen culture

Peritoneal fluid specimens in the cohort were sent directly to the laboratory or kept at 4 °C until the next day if they were collected after hours. For aerobic culture, the fluid specimens were inoculated onto Columbia blood agar and MacConkey agar without salt. The plates were incubated at 37 °C in air atmosphere and were examined 24 and 48 h after incubation.

For anaerobic culture, the fluid specimens were plated onto Columbia blood agar, neomycin blood agar, and nalidixic acid agar and each plated agar further impregnated with metronidazole discs so as to guide sensitivity analysis. All plates were incubated in an anaerobic gas jar with O_2_ levels <1 % and CO_2_ levels between 9 and 13 % and examined for growth at 24, 48, 96 and 120 h after incubation.

All aerobic isolates were fully identified. Specimens with anaerobic isolates having more than one anaerobe identified were classified as mixed anaerobe. Sensitivity analysis was conducted with the aid of a rapid and automated VITEC-2 compact system (Biomérieux, France). This system has been in place since 2005. The Clinical and Laboratory Standards Institute dilution method was used for sensitivity testing between 1998 and 2005, and before this time, the Stokes’ method of sensitivity testing was used.

### Data collection

Data recorded included: demographic data, microbiological data (peritoneal fluid specimens and susceptibility to antibiotics), antibiotic management (initial therapy, changes in therapy, and duration of treatment) and outcomes. Infectious complications were defined as those occurring within 30 days of surgery and included intra-abdominal abscess and wound infection. The intra-abdominal abscesses were confirmed by imaging and microbiological samples. Wound infection was confirmed clinically and by microbiological samples. Patients who received oral doses of antibiotics in the community within a 1-week period before hospital admission were recorded. Adequate empirical antibiotic treatment was defined as resolution of disease with initial or step-down antibiotic treatment after primary surgery. Empirical antibiotic treatment was inadequate if the infection was non-resolving and additional antibiotics were commenced post-operatively based on intraperitoneal fluid culture results.

### Data analysis

Statistical analysis was conducted using the STATA 11.0 (Stata, College Station, TX) software. Continuous data were expressed as median and percentiles (25–75 %) and analysed by a Mann–Whitney *U* test. Data expressed by percentage of children were analysed by Chi-square and Fisher’s exact test as appropriate. Differences were considered statistically significant at the 5 % level (*P* < 0.05). For subgroup analysis, data was classified into two periods: 1995–2002 (36 patients) and 2003–2008 (33 patients). This division was necessary to identify trends, if any, in the use of antibiotics.

Like previous studies, potential variables that may be associated with a higher risk of post-operative infection and hospitalisation in the cohort of patients with appendicitis-related community acquired peritonitis were examined [1, 2]. Significant risk factors identified after univariate testing (defined by *P* < 0.2) were further tested in a multivariate logistic regression model [1, 2]. Variables associated with *P* < 0.05 after multivariate analysis were considered independent factors of risk. Odds ratios and their 95 % confidence intervals were calculated.

## Results

### Patients

The 69 children with secondary appendicitis-related bacterial peritonitis included 31 females. The median age at the time of surgery was 8 (5–11) years.

### Clinical outcome

56/69 (81 %) patients had localised peritonitis and the remaining 13/69 (19 %) had generalised peritonitis. 68/69 (98.5 %) patients had open appendicectomy. Two children underwent a ‘second look’ operation in both cases for prolonged ileus in the post-operative period. 14/69 (20 %) patients had infective complications. There were 18 infective complications recorded in the 14 patients (9 intra-abdominal collections, 5 superficial wound infections, 2 deep wound infections/dehiscence and 2 chest infections). The median length of stay in hospital was 6 (5–8) days.

### Microbiology

The microorganisms identified in the peritoneal specimens of the 69 patients are shown in Table 1. Single isolates were identified in 31/69 patients (45 %), and multiple isolates were identified in the other 38 (55 %) patients. *Escherichia coli* was identified in 56/69 (81 %) specimens; Four of these isolates were resistant to amoxicillin–clavulanate and only one of the *E. coli* strains was resistant to the aminoglycoside, gentamicin. There was no isolates of extended spectrum beta lactamases or carbapenem-resistant *Enterobacteriaceae* identified in any of the specimens, but these may be important in the future. Streptococcal species (two Group F streptococci and three β-haemolytic streptococci) were identified in 5/69 (7 %) specimens. All the streptococcal species were sensitive to amoxicillin–clavulanate. *Pseudomonas aeruginosa* was identified in 4/69 (6 %) specimens; all were sensitive to gentamicin. Mixed anaerobes were identified in 37/69 (54 %) specimens; two anaerobic isolates were resistant to amoxicillin–clavulanate and one isolate was resistant to metronidazole. A resistant-sensitive “synergism” was found between metronidazole and gentamicin for anaerobes and coliforms. Simply put, we noted sensitivity to gentamicin in one case of metronidazole-resistant anaerobes and sensitivity to metronidazole was documented in one case of gentamicin-resistant coliform.

**Table 1:** Microorganisms isolated from peritoneal fluid specimens (*n* = 69)

Microorganisms	*N* (%)
Aerobes	
Gram negative	
*Escherichia coli*	56 (81)
Other *Enterobacteriaceae*	5 (7)
*Pseudomonas aeruginosa*	4 (6)
Gram positive	
Group F streptococci	2 (3)
B-haemolytic streptococci	3 (4)
Anaerobes	37 (54)

### Antibiotic treatment

All patients received antibiotics pre-operatively. Median duration of intravenous antibiotic treatment was 4 (3–6) days. 9/69 (13 %) patients had received oral antibiotics in the community prior to presentation to hospital (Amoxicillin in six cases, amoxicillin–clavulanate in two cases and erythromycin in one case). For initial in-hospital treatment, 25/69 (36 %) patients received triple drug therapy (amoxicillin–clavulanate/cephalosporin + metronidazole + aminoglycoside) and the other 44/69 (64 %) patients received a double combination of amoxicillin–clavulanate/cephalosporin and metronidazole. We were unable to assess exactly what guided these decisions. Antibiotic treatment changed based on findings at operation without peritoneal specimen culture and sensitivity results in 10/69 (14 %) children. Antibiotic treatment was considered inadequate and modified in accordance with culture results in 4/69 (6 %) children. 13/69 (19 %) patients had de-escalation of treatment following laboratory susceptibility results.

### Use of cephalosporin versus amoxicillin–clavulanate combination therapy

Over time, cephalosporin combination therapy became less frequently used as initial treatment in the treatment of secondary community acquired peritonitis. They accounted for 86 % of the empirical treatment from 1995 to 2002 and 67 % of empirical treatment from 2003 to 2008. Amoxicillin–clavulanate combination therapy became increasingly used in the treatment of appendicitis-related peritonitis as the use of cephalosporins declined. In 12 out of the 13 patients who had de-escalation of treatment following laboratory susceptibility results, this involved stopping a cephalosporin and commencing amoxicillin–clavulanate instead.

The antibiotic susceptibility data suggested that combination therapy involving amoxicillin–clavulanate and gentamicin would have been appropriate empirical treatment in 68/69 (99 %) patients. Addition of metronidazole to this regime would have provided 100 % initial empirical coverage.

### Risk factors for post-operative infection and hospital stay

Significant variables associated with increased risk of post-operative infection are presented in Table 2. Results of testing of variables that may be associated with prolonged hospitalisation are presented in Table 3. Independent risk factors for post-operative infectious complications identified on multivariate analysis were: isolation of *E. coli* resistant to amoxicillin–clavulanate in the peritoneal fluid specimen (OR, 21.88 [1.7–277.2]; *P* = 0.017) and inadequate initial antibiotic therapy (OR, 18.37 [1.1–321.0]; *P* = 0.046). Female gender (OR, 3.11 [0.7–14.4]; *P* = 0.146), isolation of *P. aeruginosa* (OR, 1.55 [0.1–2.2]; *P* = 0.7697) or finding of appendicular abscess (OR, 2.68 [0.6–11.8]; *P* = 0.193) did not reach statistical significance on multivariate analysis.

**Table 2:** Univariate analysis of factors associated with a risk of post-operative infection in children with secondary peritonitis from appendicitis

	Post-op infection (*N* = 14)	No post-op infection (*N* = 55)	*P*
Age (years), median (25th–75th)	8.5 (5–12)	8 (5–11)	0.512
Sex ratio F/M, *n*	4/10	27/28	0.168*
Generalised peritonitis, *n* (%)	3 (21)	10 (18)	0.781
Intra-abdominal abscess, *n* (%)	6 (43)	14 (25)	0.200*
Use of cefuroxime–metronidazole, *n* (%)	9 (64)	36 (65)	0.935
Use of amoxicillin–clavulanate combination, *n* (%)	4 (29)	13 (24)	0.702
Bacteriology, *n* (%)			
Monomicrobial	6 (43)	27 (49)	0.677
Polymicrobial	8 (57)	28 (51)	
*Escherichia coli*, *n* (%)	11 (79)	45 (82)	0.781
Anaerobic microorganisms, *n* (%)	8 (57)	29 (52)	0.767
*Pseudomonas aeruginosa*, *n* (%)	2 (14)	2 (3)	0.128*
Other *Enterobacteriaceae*, *n* (%)	2 (14)	3 (5)	0.255
*E. coli* strain resistant to amoxicillin–clavulanate, *n* (%)	3 (21)	1 (2)	0.005*
Streptococci spp (two Group F + three β-haemolytic streptococci), *n* (%)	0 (0)	5 (9)	0.241
Pre-hospital antibiotic treatment	7 (13)	2 (14)	0.872
Inadequate initial treatment, *n* (%)	3 (21)	1 (2)	0.005*

Comparison by* X* ^2^, Fisher exact test, Mann and Whitney test as appropriate

* Variables with a *P* value <0.2 were tested in a multivariate logistic regression model

**Table 3:** Univariate analysis of factors associated with a risk of hospitalisation length of stay above 7 days in children with secondary peritonitis from appendicitis

	>7 days (*n* = 22)	<7 days (*n* = 47)	*P*
Age (years), median (25th–75th)	8 (4–10)	9 (5–11)	0.442
Sex ratio F/M, *n*	7/15	24/23	0.134*
Generalised peritonitis, *n* (%)	6 (27)	7 (15)	0.220
Intra-abdominal abscess, *n* (%)	5 (23)	15 (32)	0.433
Use of cefuroxime–metronidazole, *n* (%)	15 (68)	30 (64)	0.724
Use of amoxicillin–clavulanate combination, *n* (%)	6 (27)	11 (23)	0.728
Bacteriology, *n* (%)			
Monomicrobial	7 (32)	21 (45)	0.069*
Polymicrobial	15 (68)	26 (55)	
*Escherichia coli*, *n* (%)	19 (86)	37 (79)	0.449
Anaerobic microorganisms, *n* (%)	15 (68)	22 (47)	0.097*
*Pseudomonas aeruginosa*, *n* (%)	1 (5)	3 (6)	0.761
Other *Enterobacteriaceae*, *n* (%)	2 (9)	3 (6)	0.686
*E.coli* strain resistant to amoxicillin–clavulanate, *n* (%)	2 (9)	2 (4)	0.423
Streptococci spp (two Group F + three β-haemolytic streptococci), *n* (%)	0 (0)	5 (11)	0.112*
Pre-hospital antibiotic treatment	7 (15)	2 (9)	0.505
Inadequate initial treatment, *n* (%)	2 (9)	2 (4)	0.423
Length of intravenous antibiotic treatment, median (25th–75th)^a^	5.5 (4.7–7)	4 (3–4.7)	0.001*

Comparison by* X* ^2^, Fisher exact test, Mann and Whitney test as appropriate

* Variables with a *P* value <0.2 were tested in a multivariate logistic regression model

^a^Included variable is a surrogate for disease severity and not a risk factor per se

We did not identify any significant risk factor for prolonged hospitalisation more than 7 days in patients with appendicitis-related peritonitis on multivariate analysis. We, however, found that the duration of hospitalisation was directly related to the duration of intravenous treatment required to treat each case based on the severity and clinical response.

## Discussion

In the peritoneal samples of 69 children with secondary peritonitis from appendicitis, *E. coli* was identified in 56/69 (81 %) peritoneal specimens; four isolates were resistant to amoxicillin–clavulanate, and one other isolate was resistant to gentamicin. Anaerobes were identified in 37/69 (54 %) peritoneal specimens; two anaerobic isolates were resistant to amoxicillin–clavulanate and one isolate was resistant to metronidazole. *P. aeruginosa* was identified in 4/69 (6 %) peritoneal specimens, and all were susceptible to gentamicin. Streptococcal species (two Group F streptococci and three β-haemolytic streptococci) were identified in 5/69 (7 %) specimens, and all were susceptible to amoxicillin–clavulanate. Post-operative infectious complications occurred in 14/69 (20 %) patients and predictors of increased post-operative complications were identified. The rationale for use of combination therapy in the treatment of secondary peritonitis was also presented.

The current study may differ from previous studies due to the homogeneity of the group studied but striking other similarities need to be underpinned. *E. coli* and *Bacteroides fragilis* (anaerobe) are the main pathogens involved in paediatric appendicitis-related peritonitis [1, 8, 9, 20]. Isolation of *E. coli* resistant to amoxicillin–clavulanate may be associated with post-operative peritonitis [1]. Appropriate initial antimicrobial therapy may predict successful treatment of peritonitis [21]. Isolation of *P. aeruginosa* in peritoneal specimens may be associated with post-appendicectomy surgical infections in the absence of appropriate primary antibiotics [4, 22].

Dumont et al. [1] evaluated microbiology and antimicrobial susceptibility of peritoneal isolates in children who underwent surgery for community acquired peritonitis in a single surgical centre. The study’s sample size was similar to that of this study and included only 70 patients: 69 children with peritonitis from appendicitis and 1 from perforation of the small intestine. They found that *E. coli* and anaerobes were the main pathogens involved in paediatric community acquired peritonitis. They found a 10.4 % resistance rate of coliforms to amoxicillin–clavulanate. Similar to the current study, they showed that the presence of *E. coli* resistant to amoxicillin–clavulanate was an independent risk factor associated with post-operative peritonitis.

Krobot et al. [21], in a multicentre study of 162 patients with perforated appendicitis, found that appropriateness of initial parenteral antibiotic therapy was a predictor of clinical success and length of stay. Similarly, they demonstrated a high risk of post-operative infections in patients with inadequate empirical treatment.

Two studies in the paediatric population had found a positive correlation between isolation of *P. aeruginosa* in peritoneal specimens and post-appendicectomy surgical infections [4, 22]. Chen et al. [4] isolated *P. aeruginosa* in 18/117 (15 %) fluid specimens of patients with appendicitis and demonstrated a positive correlation between isolation of *P. aeruginosa* and surgical site infections. They found that *P. aeruginosa* was frequently not covered by chosen prophylactic antibiotics. 7/18 (39 %) *P. aeruginosa* in that study was resistant to cefuroxime, and they identified pseudomonas in peritoneal specimens of 5/8 (63 %) patients who later developed surgical site infections. Yellin et al. [22], also reported a high rate of infectious complications in patients with appendicitis from whom *P. aeruginosa* were isolated. Compared to these two studies, this study identified *P. aeruginosa* in 4/69 (6 %) specimens and all 4 isolates were sensitive to gentamicin. We found that the four patients with *P. aeruginosa* in their peritoneal specimens had been on cefuroxime and metronidazole as empirical treatment; two of these patients subsequently developed significant intra-abdominal infection and required switch to piperacillin–tazobactam and gentamicin following drainage procedures.

Conflicting data with respect to the role of *P. aeruginosa* in the outcome of peritonitis may be explained by the lack of appropriate antibiotics in the primary treatment protocols. The summary of the data is that addition of an aminoglycoside is paramount when considering treatment for appendicitis-related peritonitis, and that inadequate initial empirical treatment may lead to post-operative infectious complications [1, 23]. Pseudomonas species are also inherently resistant to amoxicillin–clavulanate, and post-operative infections might develop if this antibiotic were to be used alone in the treatment of associated peritonitis.

Knowing the microbial and antibiotic resistance profile is critical in an attempt to provide the best empirical antibiotic treatment for secondary peritonitis arising from appendicitis in children [1]. There is no single empirical antibiotic known to reduce post-appendicectomy infectious complications in patients with complicated appendicitis [3, 8, 14, 21, 24]. The current local policies do not favour the use of cephalosporins for the treatment of infections. We found evidence to promote the continued use of amoxicillin–clavulanate and aminoglycoside, gentamicin for the treatment of secondary peritonitis due to appendicitis in children. We showed that adding amoxicillin–clavulanate to the combination of metronidazole and gentamicin as initial empirical treatment provided 100 % coverage of resistant organisms. The use of only amoxicillin–clavulanate and gentamicin was appropriate in 98.5 % of cases.

With respect to the duration of antibiotics, the median duration of intravenous antibiotic treatment was 4 days. Patients with more severe disease required at least 5 days of intravenous antibiotics and this factor contributed to prolonged hospitalisation >7 days in the cohort. In the experience of the authors, the antibiotic susceptibility reports may recommend the continuation of gentamicin which is only available intravenously. We think initial treatment in the setting of appendicitis-related peritonitis using triple antibiotic combination therapy (amoxicillin–clavulanate, metronidazole and gentamicin) is appropriate while awaiting definitive culture and sensitivity results. This may help reduce the incidence of post-operative infectious complications associated with amoxicillin-resistant *E. coli* in appendicitis-related peritonitis. In addition, other factors such as attention to basic infection control strategies, the surgeon’s experience and technique, the duration of the procedure, hospital and operating room environment, instrument sterilisation techniques, pre-operative preparation and management of any underlying medical condition of the patient should also be considered [24]. Antibiotic treatment should of course be narrowed once sensitivity results become available. In this study, 13/69 (19 %) patients had de-escalation of treatment following laboratory susceptibility results.

Retrospective, single-centre studies may limit generalisations. Further, susceptibility to cephalosporins was not routinely available due to local antimicrobial management policy and clinicians did not adhere to a strict antimicrobial protocol. However, the study findings are in line with previously documented work in this area. We feel such findings may help in the formation of consensus guidelines/design of future trials. Patients in this study were screened for risk factors for post-operative infection and length of stay. Independent risk factors for post-operative infection were inadequate initial empirical antibiotic treatment and the presence of amoxicillin-resistant *E. coli*. Rationale for adding other empirical antibiotics to amoxicillin–clavulanate in the treatment of appendicitis-related peritonitis in children has been presented.

## Conclusion

Perforation of the appendix inevitably leads to significant bacterial contamination and morbidity. *E.coli* and mixed anaerobes are the predominant organisms involved in the resulting peritonitis. No single antimicrobial treatment is effective and antibiotic resistance is common. Inadequate initial empirical antibiotic and amoxicillin–clavulanate resistant *E. coli* may contribute to increased post-operative infectious complications. Based on the clinical data presented, a triple antibiotic combination of amoxicillin–clavulanate, gentamicin and metronidazole is reasonable empiric basis for treatment of appendicitis-related peritonitis.
